# Targeting neutrophil extracellular traps: an emerging strategy for improving the management of refractory asthma

**DOI:** 10.3389/fmed.2026.1780034

**Published:** 2026-03-26

**Authors:** Xue-hui Wang, Mei-zhen Song, Meng Li

**Affiliations:** 1 Department of Respiratory Medicine, First Affiliated Hospital of Heilongjiang University of Chinese Medicine, Harbin, China; 2 Graduate School of Heilongjiang University of Chinese Medicine, Harbin, China; 3 Heilongjiang Provincial Traditional Chinese Medicine Data Center, First Affiliated Hospital of Heilongjiang University of Chinese Medicine, Harbin, China

**Keywords:** biomarkers, cost-effectiveness analysis, neutrophil extracellular traps, neutrophilic phenotype, precision medicine, refractory asthma, targeted therapy

## Abstract

Treatment-refractory asthma, particularly the neutrophilic-predominant phenotype, poses a significant clinical challenge. Current therapies, especially corticosteroids, have limited efficacy in controlling inflammation in these patients, highlighting the need for novel targets against non-eosinophilic inflammation. This review examines the central role of neutrophil extracellular traps (NETs) in driving persistent airway inflammation, tissue damage, and remodeling in refractory asthma. It also evaluates preclinical and translational evidence for therapeutic strategies targeting NETs—such as inhibiting their formation, promoting degradation, neutralizing toxic components, and enhancing clearance. Despite challenges in target specificity, patient heterogeneity, and delivery, integrating these strategies with precision medicine could open new disease-modifying avenues to improve outcomes in treatment-refractory asthma, especially the neutrophilic phenotype.

## 1 Introduction

Treatment-refractory asthma is a severe subtype characterized by persistent symptoms and frequent exacerbations. This occurs despite high-dose inhaled corticosteroids combined with long-acting β2-agonists, good adherence, and the exclusion of confounders like poor inhaler technique, comorbidities, or environmental exposures (1, 2). Clinically, it presents with persistent airflow limitation, recurrent acute attacks, and often a dependence on systemic corticosteroids (1, 3). This leads to a substantial burden on both patient quality of life and healthcare systems. Epidemiological studies show that although refractory asthma affects only 5–10% of asthma patients, it accounts for more than 50% of asthma-related healthcare costs (3). This highlights its disproportionate socioeconomic impact and the unmet clinical need.

Current therapeutic strategies for refractory asthma have limited efficacy. This is particularly true for the inflammatory phenotype dominated by neutrophil infiltration (4). Corticosteroids, the cornerstone of conventional anti-inflammatory therapy, effectively suppress eosinophilic inflammation but are largely ineffective against neutrophilic inflammation (5). In some cases, corticosteroids may even prolong neutrophil survival, which could potentially worsen the inflammatory response (6). This therapeutic gap underscores that the pathophysiology of refractory asthma—especially the neutrophilic subtype—involves mechanisms more complex than the classic Th2 pathway (7). It necessitates novel therapeutic targets against non-eosinophilic inflammatory pathways.

Neutrophil extracellular traps (NETs) are web-like structures released by activated neutrophils. They consist of chromatin deoxyribonucleic acid (DNA) decorated with granular proteases like myeloperoxidase and neutrophil elastase (8). Initially recognized as a key innate immune defense mechanism for trapping and killing pathogens, emerging evidence shows that NETs formation and release act as a double-edged sword (8). In chronic inflammatory diseases, excessive or dysregulated NETs release can directly damage airway epithelial and endothelial cells (9). This occurs through associated proteases and reactive oxygen species, which amplify inflammatory cascades, worsen tissue injury and mucus hypersecretion, and may disrupt immune homeostasis. This process can sustain a vicious cycle of inflammation. Elevated NETs components are found in the airways and sputum of patients with severe or refractory asthma (8). Their levels correlate with the degree of airflow obstruction and neutrophilic inflammation (10).

Given the limitations of current therapies and the potential role of NETs in driving persistent airway inflammation, this perspective article scrutinizes targeting NETs as an emerging strategy for improving refractory asthma management. We examine the theoretical basis of how NETs contribute to and exacerbate refractory asthma pathology. We also review current research on interventions targeting NETs formation, components, and downstream effects. This provides a forward-looking perspective for developing novel treatments to overcome current therapeutic limitations. Establishing a clear mechanistic understanding of how NETs drive airway inflammation and structural remodeling is therefore essential for informing the targeted therapeutic strategies discussed in the following section.

## 2 Central role of NETs in the pathogenesis of treatment-refractory asthma

**Figure 1:**
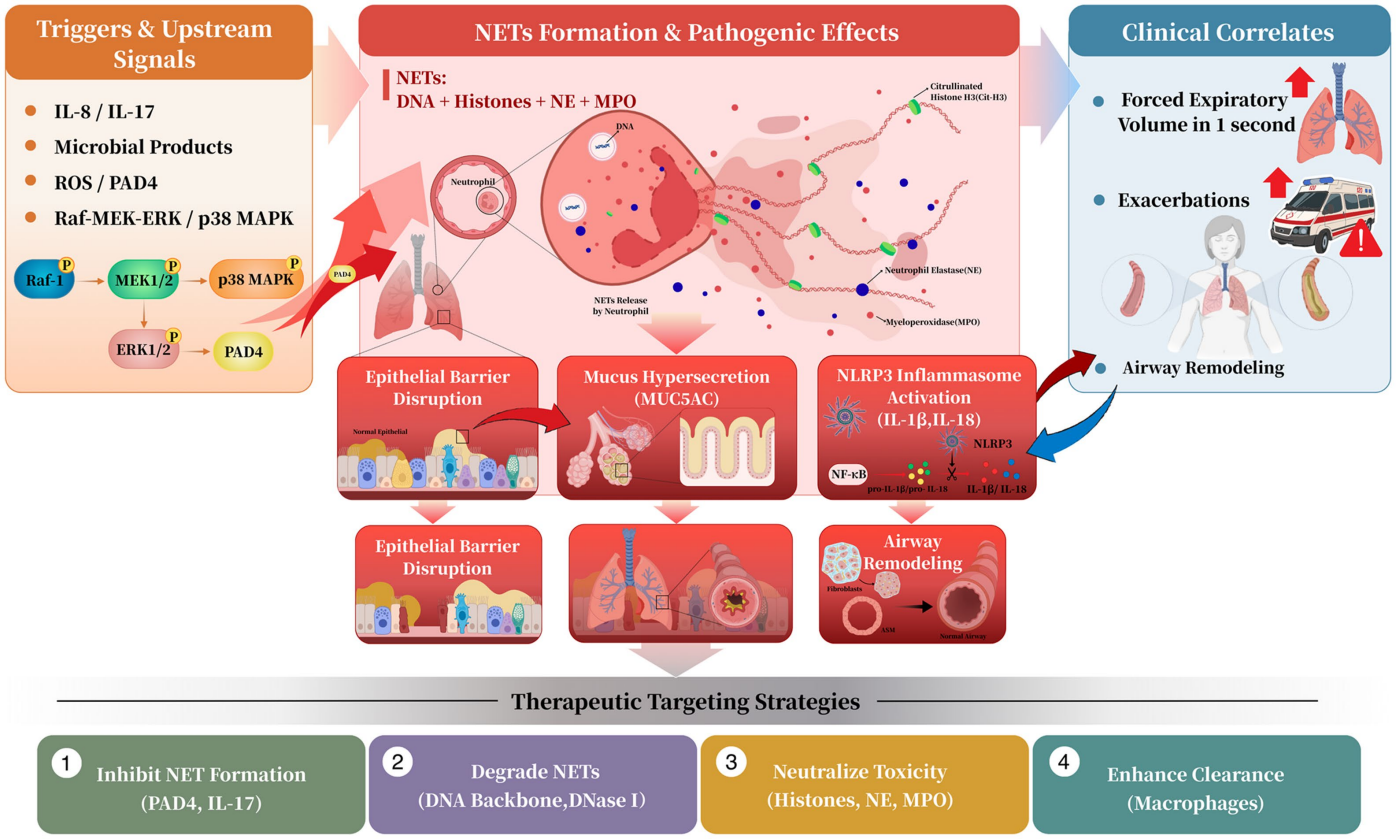
Central role of NETs in the pathogenesis and therapeutic targeting of treatment-refractory asthma. Schematic overview illustrating NET formation, airway epithelial injury, inflammatory amplification, and major therapeutic strategies targeting NET biology in treatment-refractory asthma. Created with BioRender.com.

NETs are central to the pathogenesis of treatment-refractory asthma (Figure 1). They contribute not only to acute defense responses but also to sustained inflammation, tissue damage, and chronic pathological processes such as airway remodeling.

### 2.1 Formation and regulation of NETs in refractory asthma

NETs formation is significantly upregulated in the airways of patients with refractory asthma (11). This process is driven by specific inflammatory signals, including cytokines such as interleukin-8 (IL-8) and interleukin-17 (IL-17), along with lower respiratory tract microorganisms and their byproducts (12). These stimuli activate key signaling pathways, including Raf–MEK–ERK and p38 MAPK, and depend on reactive oxygen species generation and peptidylarginine deiminase 4 (PAD4) activity (13, 14). These events lead to chromatin decondensation and NETs release, establishing the basis for excessive NETs production in the airways.

### 2.2 Direct tissue-damaging effects of NETs

Released NETs directly damage airway tissues through their cytotoxic components. The histone backbone of NETs is inherently cytotoxic and can disrupt airway epithelial cell membranes, compromising epithelial barrier integrity (15). Simultaneously, NETs-embedded serine proteases, such as neutrophil elastase, degrade epithelial tight junction proteins, worsening barrier dysfunction (16). This breach exposes airways to more environmental irritants and allergens. The released damage-associated molecular patterns also act as alarmins, directly stimulating sensory nerve endings and enhancing airway hyperresponsiveness (10). Moreover, components like neutrophil elastase are potent mucin secretagogues that upregulate mucin MUC5AC expression and secretion, driving the mucus hypersecretion and plug formation common in refractory asthma (17).

### 2.3 Amplification of inflammatory circuits by NETs

NETs also amplify and sustain inflammatory circuits through multiple mechanisms. First, NETs components, such as myeloperoxidase (MPO) and double-stranded DNA (dsDNA), can act as autoantigens (18). This may trigger autoantibody production (e.g., anti-MPO, anti-dsDNA), linking innate immune responses to adaptive immune dysregulation. Second, NETs can activate inflammasomes, particularly the NLRP3 inflammasome, in immune cells such as macrophages (19–21). This leads to the release of potent pro-inflammatory cytokines, including interleukin-1β (IL-1β) and interleukin-18, creating a self-reinforcing cycle of chronic inflammation. Finally, NETs and their components interact with airway smooth muscle cells and fibroblasts, stimulating proliferation and collagen synthesis (9, 19). This suggests a potential mechanism for NETs involvement in the airway remodeling characteristic of refractory asthma. These interconnected pathogenic processes are schematically summarized in Supplementary Figure 1 to provide an integrated visualization of NETs-driven disease progression.

### 2.4 Clinical evidence linking NETs to disease severity

Clinical evidence strongly supports the relevance of these mechanisms. Elevated NETs markers—such as cell-free DNA (cfDNA), MPO-DNA, and citrullinated histone H3—are consistently detected in sputum, bronchoalveolar lavage fluid, serum, and airway biopsies from patients with refractory asthma, especially those with a neutrophilic phenotype (22). Critically, these marker levels correlate positively with clinical indicators, including reduced forced expiratory volume in 1 s, worsened asthma control questionnaire scores, and sputum neutrophil percentages (23). These markers often increase further during acute exacerbations. This correlative evidence bridges experimental findings with clinical disease, establishing NETs not only as disease activity biomarkers but also as key effectors in the progression of refractory asthma (19, 24).

Importantly, NETs should not be viewed as a single unifying driver of neutrophilic asthma. Instead, they function as a critical integrative component within a broader inflammatory network (25). Cytokine pathways—such as IL-17 signaling, inflammasome activation, and microbial stimuli—interact bidirectionally with NETs formation (26). In this context, NETs serve as an amplification node, linking innate immune activation, epithelial damage, and sustained neutrophilic inflammation (9). This mechanism reinforces, but does not replace, other pathogenic processes contributing to disease heterogeneity in treatment-refractory asthma.

Collectively, these mechanistic insights elucidate the multifaceted role of NETs in sustaining refractory airway inflammation and reveal multiple intervention points across the NETs lifecycle. Accordingly, therapeutic strategies targeting NETs formation, persistence, and downstream inflammatory effects emerge as a logical extension of these pathogenic findings. This mechanistic understanding thus provides the conceptual foundation linking disease pathology to therapeutic innovation.

## 3 Therapeutic strategies targeting NETs: from concept to practice

Therapeutic strategies targeting NETs have progressively evolved from conceptual hypotheses into research domains characterized by increasingly well-defined molecular targets and intervention pathways. These strategies are grounded in the lifecycle and pathogenic mechanisms of NETs, enabling therapeutic modulation at multiple stages of NETs formation, persistence, and downstream inflammatory activity (27). Current evidence supporting NETs-targeted interventions spans multiple experimental levels, including *in vitro* mechanistic investigations, *in vivo* animal models, and emerging clinical observations (28). Recognizing the distinctions among these evidence tiers is critical for evaluating translational readiness, as therapeutic approaches demonstrating promising effects in experimental systems have not yet been consistently validated in large-scale human studies (29, 30). Accordingly, NETs-directed therapeutic development can be broadly categorized into four primary approaches: inhibiting formation, promoting degradation, neutralizing toxicity, and enhancing clearance (Table 1; Figure 1). Importantly, these therapeutic categories map directly onto the distinct stages of NETs biology outlined above, thereby translating mechanistic insights into a structured framework for intervention.

**Table 1:** Therapeutic strategies targeting NETs in treatment-refractory asthma.

Strategy category	Target/mechanism	Representative agents	Evidence level	Key notes
Inhibit formation	PAD4, IL-17, ROS	GSK484, Cl-amidine	Preclinical	Risk of infection and potential impairment of antimicrobial defense (8, 31–36)
Degrade NETs	DNA backbone	DNase I	Preclinical/translational	Improves mucus properties and airway clearance (39–43)
Neutralize toxicity	Histones, NE, MPO	Heparin, sivelestat	Preclinical/early clinical	Local delivery preferred to reduce systemic effects (44–52)
Enhance clearance	Macrophage efferocytosis	IL-10, CD44 modulation	Experimental	Emerging therapeutic concept requiring further validation (56–63)
Drug repurposing	Multi-target	Azithromycin, clopidogrel	Observational/preclinical	Cost-effective strategy with translational potential (65–67)

NETs, neutrophil extracellular traps; PAD4, peptidylarginine deiminase 4; IL-17, interleukin-17; ROS, reactive oxygen species; DNase I, deoxyribonuclease I; NE, neutrophil elastase; MPO, myeloperoxidase; IL-10, interleukin-10; CD44, cluster of differentiation 44.

### 3.1 Strategy 1: inhibiting NETs formation

Inhibiting NETs formation is the most direct interventional strategy. This focuses on blocking key molecules in NETosis. One method targets essential enzymes, particularly PAD4 (8). PAD4 catalyzes histone citrullination, initiating chromatin decondensation. PAD4 inhibitors such as GSK484 and Cl-amidine have reduced airway NETs formation and improved inflammation and hyperresponsiveness in asthma animal models, demonstrating promising preclinical potential (8, 31). However, most investigations of PAD4 inhibition have been conducted in murine asthma models and cellular experiments, while clinical evidence derived from human populations remains limited (32). An important concern associated with this strategy is that excessive suppression of NETs formation may interfere with physiological antimicrobial defense mechanisms, potentially increasing susceptibility to respiratory infections (33–36). Furthermore, discrepancies between controlled experimental inflammatory models and the biological heterogeneity of human asthma phenotypes may constrain the direct translation and generalizability of current findings to clinical practice (32).

Beyond direct enzymatic inhibition, alternative approaches aim to modulate upstream signaling pathways that regulate NETs generation. For example, monoclonal antibodies against IL-17 or IL-8 can reduce neutrophil activation and NETs release by neutralizing these stimulatory signals (37, 38). Additionally, Rho-associated coiled-coil kinase inhibitors may serve as an upstream intervention by influencing NETosis through cytoskeletal reorganization (31). Collectively, these approaches highlight the therapeutic potential of preventing NETs formation at early stages of the inflammatory cascade while underscoring the need to balance anti-inflammatory efficacy with preservation of host immune defense.

### 3.2 Strategy 2: degrading or destabilizing formed NETs

For NETs that have already formed, strategies aim to degrade their structure or neutralize their toxic components. One method uses DNase I, which hydrolyzes the DNA backbone of NETs, disrupting their web-like structure (39). Studies show that nebulized DNase I can effectively degrade airway NETs, improving mucus properties and airway patency (39, 40). Evidence supporting DNase-based NETs degradation originates from both preclinical models of airway inflammation and limited clinical applications in respiratory diseases, including cystic fibrosis and acute respiratory distress syndrome, suggesting potential translational relevance for NETs-driven airway disorders (40, 41). However, excessive or uncontrolled NETs degradation may inadvertently release embedded proteases, histones, and other pro-inflammatory mediators, which under certain pathological conditions could paradoxically aggravate tissue injury and inflammatory responses (42, 43).

Beyond enzymatic degradation, alternative strategies focus on neutralizing cytotoxic NETs components. Histones, which constitute a major structural and toxic element of NETs, exert strong membrane-disruptive effects on airway epithelial cells (44). Heparin can bind to positively charged histones through electrostatic interactions, thereby attenuating their cytotoxic activity (45). Similarly, anti-histone antibodies have been shown to directly block histone-mediated cellular injury (46). Both approaches have demonstrated protective effects in experimental models of lung injury, highlighting the therapeutic potential of destabilizing pathogenic NETs structures while limiting downstream tissue damage (47).

### 3.3 Strategy 3: neutralizing NETs-associated toxic components

Directly neutralizing toxic proteins carried by NETs is another important strategy. Neutrophil elastase and MPO are two major destructive enzymes on NETs (48). Oral or inhaled NE inhibitors (e.g., sivelestat) and MPO inhibitors are under clinical evaluation for various lung diseases (49, 50). Their therapeutic potential primarily lies in reducing NET-mediated proteolytic and oxidative damage to airway epithelial structures while attenuating downstream inflammatory signaling.

Most investigations into neutrophil elastase and MPO inhibition have been conducted in experimental lung injury models and early-phase clinical trials, whereas evidence derived from asthma-specific randomized controlled studies remains limited (51, 52). Current limitations include the incomplete suppression of NETs-associated inflammatory cascades and persistent residual inflammation despite enzymatic inhibition (52). In addition, uncertainties regarding optimal dosing strategies, treatment duration, and long-term safety profiles present important challenges for applying these approaches in chronic airway diseases such as refractory asthma (52, 53).

Beyond enzymatic neutralization, NETs are also known to activate downstream inflammatory pathways, particularly the NLRP3 inflammasome (54). Therefore, small-molecule NLRP3 inhibitors may provide an additional therapeutic layer by interrupting NETs-induced inflammasome activation and reducing the release of pro-inflammatory cytokines such as IL-1β (54, 55). Collectively, these approaches highlight a strategy focused on mitigating the pathological consequences of NETs while preserving certain physiological immune functions, thereby offering a potentially balanced therapeutic framework for managing NETs-driven airway inflammation. A detailed schematic overview of these therapeutic intervention pathways is provided in Supplementary Figure 2.

### 3.4 Strategy 4: enhancing NETs clearance

Enhancing the body’s clearance of NETs represents a relatively emerging therapeutic concept aimed at restoring endogenous mechanisms that limit excessive inflammatory accumulation. Macrophages play a central role in NET removal through phagocytic and efferocytotic processes; however, their clearance capacity may become impaired under persistent inflammatory conditions, thereby contributing to NET persistence and sustained airway inflammation (56, 57). Boosting their phagocytic capacity—by modulating surface receptors like CD44 or scavenger receptors, or using cytokines such as IL-10—could help restore this endogenous clearance and prevent NETs accumulation (58–60).

Despite these promising conceptual approaches, strategies designed to enhance NETs clearance remain largely at a theoretical or early experimental stage, with most supporting evidence derived from mechanistic immunology studies rather than disease-specific clinical investigations (61, 62). Important translational challenges persist, including substantial inter-patient variability in macrophage functional capacity under inflammatory conditions and the current absence of validated pharmacological agents specifically developed to promote NETs efferocytosis (61–63). These limitations highlight the need for further translational research to define clinically actionable targets and to determine whether enhancing NETs clearance can achieve sustained therapeutic benefits in treatment-refractory asthma (25, 64).

### 3.5 Drug repurposing: existing drugs with NETs-modulating potential

Several existing drugs show potential for repurposing due to their effects on NETs. For instance, macrolide antibiotics like azithromycin have immunomodulatory functions that can inhibit neutrophil activation and NETs formation (65). Heparin, beyond its anticoagulant role, can neutralize histones (66). Thienopyridine antiplatelet drugs like clopidogrel have also been reported to inhibit PAD4 activity and NETosis (67). Re-evaluating these drugs for NETs modulation may accelerate clinical translation.

In summary, therapeutic strategies targeting NETs form a multi-level, multi-target toolkit. The future challenge is to identify the dominant role of NETs in different patient subgroups and select or combine strategies accordingly for personalized treatment of refractory asthma. Despite growing mechanistic and experimental support, the clinical translation of these therapeutic approaches remains constrained by several important methodological and translational barriers, which are discussed in the following section. Future progress should depend on identifying the relative contribution of NETs-driven pathology across distinct patient subgroups and rationally selecting or combining therapeutic strategies to enable personalized management of treatment-refractory asthma.

## 4 Challenges and future directions

While the therapeutic strategies discussed above exhibit promising mechanistic rationale and encouraging preclinical evidence, their successful translation into clinical practice ultimately depends on addressing several critical methodological, translational, and implementation challenges (68–70). Although targeting NETs is a promising therapeutic strategy, its clinical translation faces multiple challenges. These challenges simultaneously delineate important priorities for future investigation and clinical development. Thus, future advances in NETs-targeted therapy will rely on the integration of mechanistic biology with biomarker-guided patient stratification and clinically adaptable therapeutic design, forming a unified translational framework capable of bridging experimental discoveries with real-world clinical implementation.

### 4.1 Translational medicine challenges

Developing effective and safe NETs-targeted therapies requires resolving several key translational issues. The first is achieving target specificity. NETs play a vital physiological role in anti-infective defense, especially in the lungs (8). Therefore, interventions should precisely distinguish pathological NETosis from beneficial immune responses to avoid compromising antimicrobial immunity (8). A central translational concern arising from this essential role of NETs in antimicrobial defense, particularly within the airways (71). Prolonged or systemic suppression of NETs may therefore raise the risk of respiratory infections (71). This underscores the need to balance anti-inflammatory benefits with preserved immune protection (72). Proposed strategies to address this challenge and mitigate infection risk include developing “smart” inhibitors activated only in inflammatory microenvironments, using localized administration (e.g., inhalation) to preserve systemic immunity, and restricting treatment to patients with clearly documented overactive NETs (34). From a precision medicine perspective, patient stratification based on NETs activity should prioritize biomarkers that are clinically accessible and analytically robust. At present, measurements such as sputum extracellular DNA and MPO–DNA complexes represent relatively feasible candidates (25, 73), whereas other markers, including citrullinated histone variants or multi-omics NETs signatures, remain largely investigational (74, 75). The second issue is disease heterogeneity. Treatment-refractory asthma is heterogeneous; not all patients have NETs-driven pathology as a core mechanism (76, 77). Future efforts should use multi-omics technologies (e.g., proteomics, metabolomics) and clinical data to identify “high-NETs phenotype” subgroups (78). These may include neutrophilic asthma patients with persistently elevated NETs markers (e.g., MPO-DNA complexes) and poor responses to existing biologics (25). This will enable precise patient selection for targeted therapy. Finally, determining the optimal timing and route of administration is essential. Key questions include whether to intervene during acute exacerbations or stable periods, and whether inhaled delivery achieves effective local concentrations with minimal systemic exposure (34, 77). These questions require systematic preclinical pharmacokinetic and pharmacodynamic studies.

### 4.2 Methodological outlook

Overcoming these challenges requires methodological innovation. First, more reliable biomarkers are needed to detect and quantify NETs *in vivo*. Current markers (e.g., cfDNA, MPO-DNA) have limitations in specificity and quantitative standardization (8). Distinguishing clinically viable biomarkers from experimental NETs profiles is critical for practical trial design and for preventing the premature extension of precision medicine frameworks. Consequently, future work should identify specific NETs “fingerprints” (e.g., citrullinated histone variants) and develop sensitive detection techniques for clinical samples (e.g., exhaled breath condensate) (79). Second, leveraging advanced models for mechanistic studies is crucial. Human airway organoid co-culture systems can model interactions between NETs and the airway barrier (80). Genetically modified animal models with asthma-associated variants can help dissect NETs formation drivers and effects, providing a foundation for target validation (81).

### 4.3 Clinical development prospects

From a clinical perspective, NETs-targeting strategies offer two major prospects. The first is combination therapy with existing biologics. For patients with mixed eosinophilic and neutrophilic inflammation, combining IL-5/IL-5R pathway inhibitors with NETs inhibitors may yield synergistic anti-inflammatory effects (25). Combining NETs-targeted therapies with biologics like dupilumab (an IL-4Rα inhibitor) is also worth exploring (8). The second prospect is designing precision clinical trials. Future trials should focus on “high-NETs phenotype” patients using enrichment designs (82). Beyond traditional endpoints, these trials should include NETs biomarker dynamics, exacerbation frequency (especially non-infectious), and patient-reported outcomes (83). Addressing these challenges will help realize the potential of NETs-targeted therapies for treatment-refractory asthma, particularly the neutrophilic phenotype.

## 5 Summary

In summary, NETs are central to the pathology of treatment-refractory asthma, especially in neutrophilic-predominant phenotypes. Their excessive formation and dysregulation directly cause airway epithelial injury, mucus hypersecretion, and airway hyperresponsiveness. They also drive disease progression and treatment resistance through multiple mechanisms. These mechanisms include activating adaptive immunity, amplifying inflammatory cascades, and potentially promoting airway remodeling.

Therefore, therapeutic strategies that target NETs—by inhibiting their formation, promoting their clearance, or neutralizing their toxic components—could modulate the disease process at its source. This represents a promising new therapeutic direction with disease-modifying potential. However, translating these strategies to the clinic faces challenges, including target specificity, patient heterogeneity, and optimal delivery methods.

Future research should therefore focus on: developing specific NETs-related biomarkers for patient stratification; optimizing targeted drug delivery to balance efficacy and safety; and designing rigorous clinical trials to explore combination therapies. This multidimensional, precision-focused research is essential for integrating NETs-targeted strategies into the management of treatment-refractory asthma and improving outcomes for this severe patient population.

## Data Availability

The original contributions presented in the study are included in the article/Supplementary material, further inquiries can be directed to the corresponding author.
