# Supplementary figures and images for: Targeting neutrophil extracellular traps: an emerging strategy for improving the management of refractory asthma
Source: Front Med (Lausanne). 2026 Mar 26;13:1780034. doi: 10.3389/fmed.2026.1780034 (PMC13063380; doi:10.3389/fmed.2026.1780034)

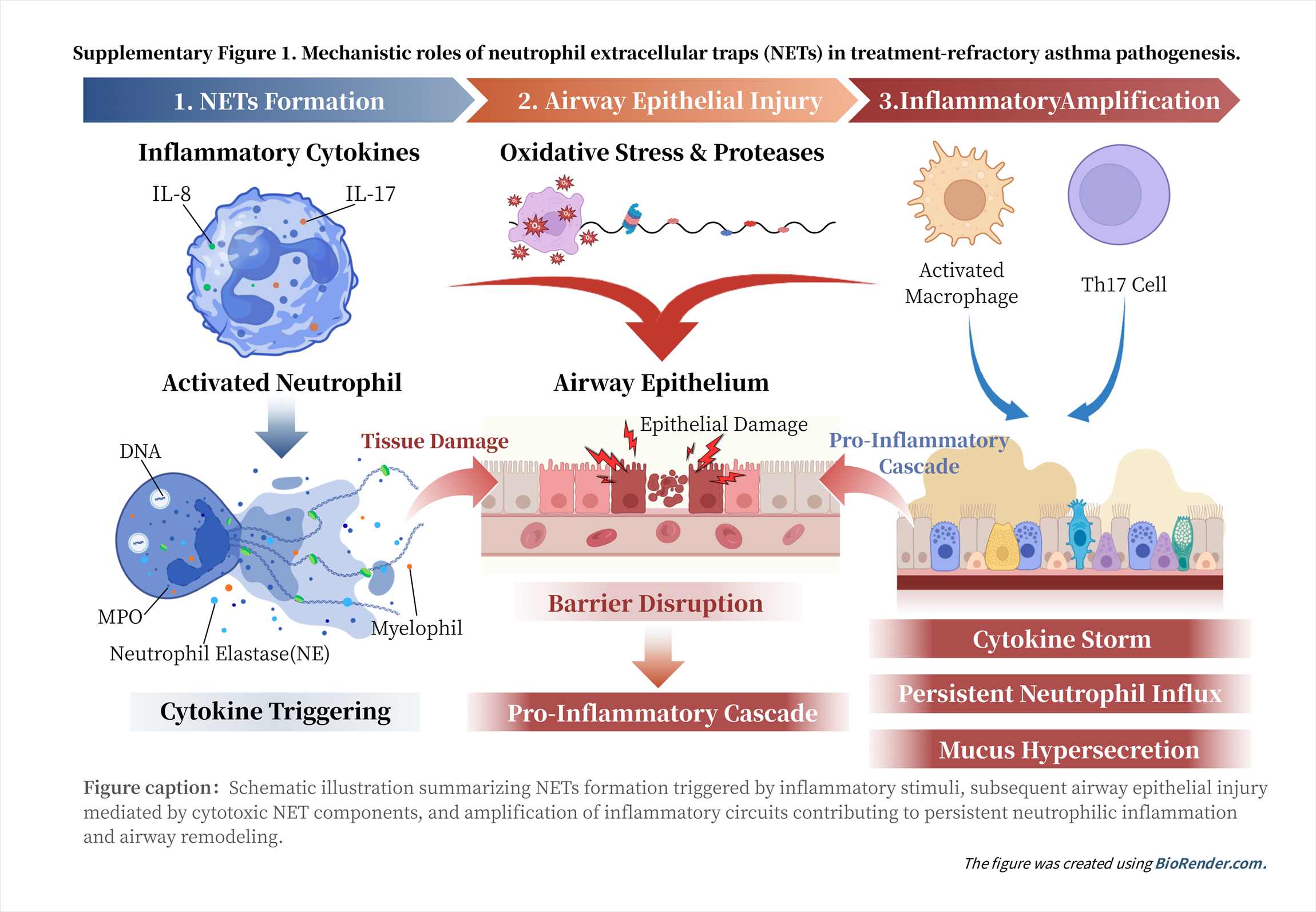

Supplement: Supplementary file 1 [file Image_1.tif]

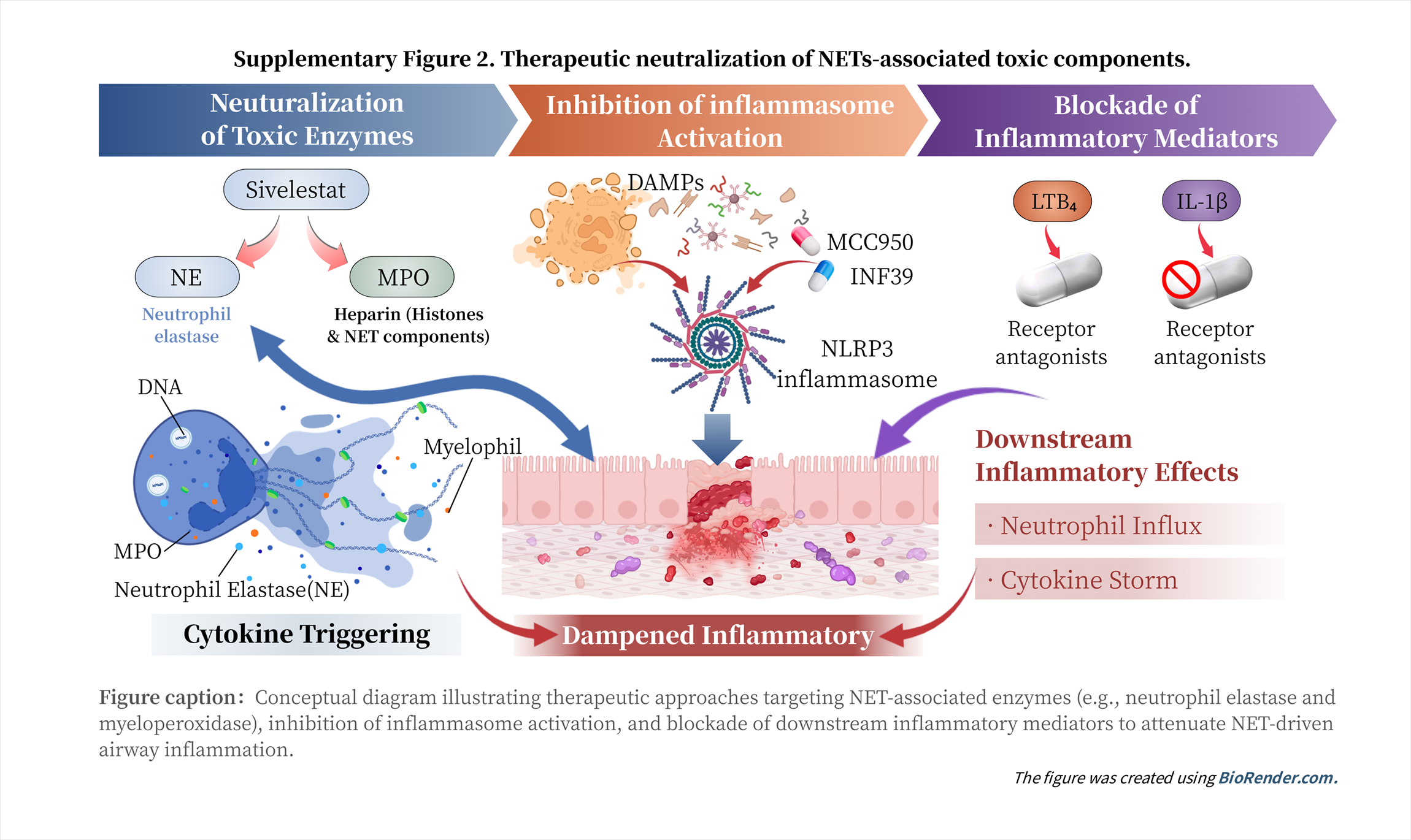

Supplement: Supplementary file 2 [file Image_2.tif]
